# SARS-CoV-2 ORF8 does not function in the nucleus as a histone mimic

**DOI:** 10.1093/procel/pwad042

**Published:** 2023-07-14

**Authors:** Ping Liu, Junjie Hu, Lei Wang

**Affiliations:** National Laboratory of Biomacromolecules, CAS Center for Excellence in Biomacromolecules, Institute of Biophysics, Chinese Academy of Sciences, Beijing 100101, China; National Laboratory of Biomacromolecules, CAS Center for Excellence in Biomacromolecules, Institute of Biophysics, Chinese Academy of Sciences, Beijing 100101, China; College of Life Sciences, University of Chinese Academy of Sciences, Beijing 100049, China; National Laboratory of Biomacromolecules, CAS Center for Excellence in Biomacromolecules, Institute of Biophysics, Chinese Academy of Sciences, Beijing 100101, China; College of Life Sciences, University of Chinese Academy of Sciences, Beijing 100049, China

Disruption of epigenetic regulation in host cells is a common evasion strategy used by viruses (Ozturkler and Kalkan 2021). Recently, Kee *et al.* (2022) reported that accessory protein ORF8 of SARS-CoV-2 can diffuse into the cell nucleus and mimic histone H3 through the ARKS motif, thereby interfering with H3 post-translational modifications (PTMs), disrupting gene transcription, and diminishing the antiviral response. These observations are surprising and unlikely because we and others have reported that SARS-CoV-2 ORF8 contains an N-terminal signal peptide for endoplasmic reticulum (ER) import (Flower *et al*. 2021; Liu *et al*. 2022), making it nearly impossible for it to wander into the nucleus. Instead, we showed that ORF8 protein accumulates in the ER lumen and escapes the degradation system by forming mixed disulfide complexes with ER-resident oxidoreductases, which subsequently activates the unfolded protein response (UPR) and remodels the ER to facilitate viral replication (Liu *et al*. 2022).

The outer nuclear membrane is part of the peripheral ER, and the perinuclear space is continuous with the ER lumen (Alberts *et al*. 2015); therefore, one should be extra careful when determining the nuclear localization of an ER-resident protein, especially when it is a soluble, luminal protein. To verify the precise localization of ORF8, we transfected cells with wild-type (WT) ORF8 and its mutant with a deletion of the ARKS motif, ORF8^∆ARKSAP^, as described by Kee *et al*. (2022). Consistently (Liu *et al*. 2022), both WT ORF8 and ORF8^∆ARKSAP^ completely co-localized with the ER marker protein disulfide isomerase (PDI) (Fig. 1A). Using super-resolution microscopy with an Airyscan detector, we observed that the ORF8 protein signals were clearly distinguished from those of the nuclear lamina marker lamin B1, even though they appeared in close proximity around the nuclear envelope (Fig. 1B and 1C). Next, we revisited the subcellular fractionation assay to investigate whether ORF8 protein enters the nucleus. We noted that the nuclear fraction could easily be contaminated with ER components, such as ER membrane protein calnexin (Fig. 1D), likely due to the continuity between the two organelles. Importantly, neither ORF8 nor ORF8^∆ARKSAP^ was detectable in the nuclear fraction under our experimental conditions (Fig. 1D), even though these proteins were sufficiently expressed in cells. Similar experiments were performed by Kee *et al*. In the case of immunofluorescence, similar results were obtained, but Kee *et al*. interpreted the proximity to lamins as a sign of ORF8 nuclear localization. In the case of cell fractionation, Kee *et al*. used a cytosolic marker protein, GAPDH, rather than an ER marker, for comparison [extended data fig. 2a and g in Kee *et al*. (2022)]. Therefore, it is impossible to assess the extent of ER contamination in the nuclear fraction, which would simply explain the appearance of ORF8 in those fractions. Taken together, ORF8 is unlikely to be a nuclear protein, but rather an ER luminal protein as described previously (Liu *et al*. 2022) (Fig. 1E). Even if some ORF8 manages to escape ER targeting, is retained in the cytosol, and subsequently leaks into the nucleus, the proportion would be extremely small.

**Figure 1.**
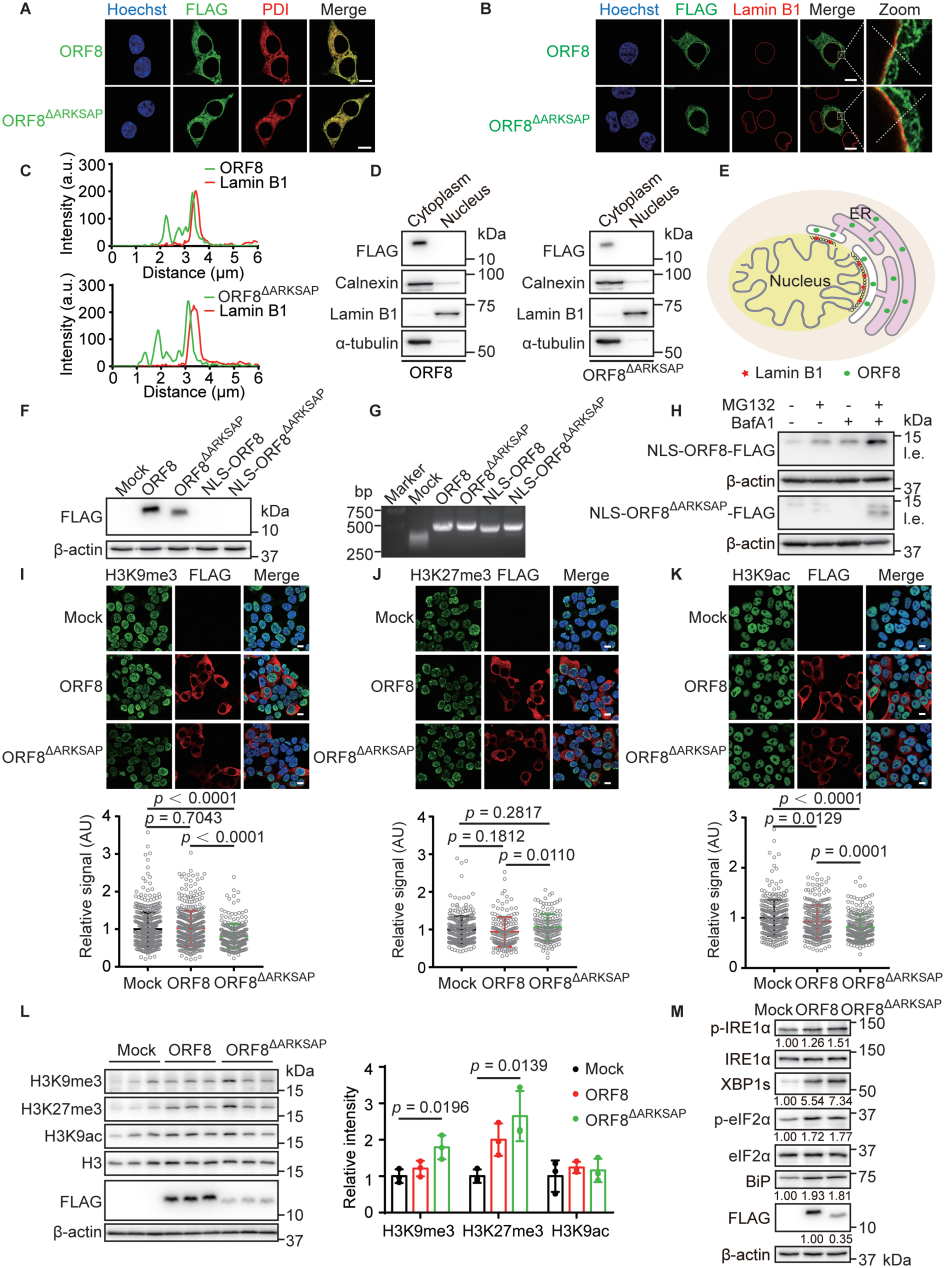
ORF8 localization and its effects on histone PTMs and UPR signaling. (A and B) Immunofluorescent analysis of ORF8 localization. HEK293T cells expressing ORF8-FLAG or ORF8^∆ARKSAP^-FLAG were immunostained using antibodies against FLAG, PDI (ER marker), and lamin B1 (nuclear lamina marker). Scale bars, 10 μm. (C) Signal intensity of ORF8 and lamin B1 enrichment throughout the cell plotted along the dotted lines in the images shown in (B). (D) Subcellular fractionation of HEK293T cells expressing ORF8-FLAG or ORF8^∆ARKSAP^-FLAG indicates that ORF8 and ORF8^∆ARKSAP^ are not present in the nucleus. Calnexin, lamin B1, and α-tubulin were used as markers of the ER, nucleus, and cytosol, respectively. (E) Illustration of the subcellular localization of ORF8. (F) Immunoblotting analysis of the protein expression of ORF8 and its mutants in HEK293T cells. (G) Analysis of mRNA levels of ORF8 and its mutants in HEK293T cells. (H) Immunoblotting of NLS-ORF8 and NLS-ORF8^∆ARKSAP^ in HEK293T cells treated with 100 nmol/L BafA1 or 8 μmol/L MG132 for 12 h. l.e., long exposure. (I–K) Immunofluorescent analysis of H3K9me3, H3K27me3, and H3K9ac in HEK293T cells expressing ORF8-FLAG or ORF8^∆ARKSAP^-FLAG. Quantitative comparison of the nuclear signal intensity is shown with a scatter dot plot analyzed by ImageJ. Scale bars, 10 μm. *n* = 522 (Mock), 445 (ORF8), and 255 (ORF8^∆ARKSAP^) cells for H3K9me3; *n* = 300 (Mock), 165 (ORF8), and 164 (ORF8^∆ARKSAP^) cells for H3K27me3; *n* = 303 (Mock), 291 (ORF8), and 254 (ORF8^∆ARKSAP^) cells for H3K9ac. (L) Immunoblotting of H3K9me3, H3K27me3, and H3K9ac in HEK293T cells expressing ORF8-FLAG or ORF8^∆ARKSAP^-FLAG. (M) Detection of the activation of IRE1α and PERK pathways by immunoblotting in HEK293T cells expressing ORF8-FLAG or ORF8^∆ARKSAP^-FLAG. The protein band intensities were quantified and normalized to β-actin intensities.

Next, we evaluated whether ORF8 could be a potent histone mimic, assuming that it somehow enters the nucleus. To this end, the signal sequence of ORF8 was replaced by the nuclear localization sequence (NLS) of SV40 large T-antigen. Surprisingly, protein expression of NLS-ORF8 and NLS-ORF8^∆ARKSAP^ was hardly detected in cells (Fig. 1F). This was less likely due to insufficient transcription, as the mRNA levels of both ER and nuclear ORF8 were similar (Fig. 1G). We have shown that ORF8 forms intermolecular mixed disulfide bonds to escape ER degradation, and a cysteine-less mutant of ORF8 was much less stable than WT ORF8 (Liu *et al*. 2022). As the environment of the cytosol and nucleus is much more reducing than that of the ER lumen (Go and Jones 2008; Wang and Wang 2023), we speculated that NLS-ORF8 is not stable in the nucleus due to the lack of mixed disulfide bond formation. Proteasome inhibitor MG132 and lysosomal inhibitor bafilomycin A1 (BafA1) increased the protein levels of NLS-ORF8 and, to a lesser extent, NLS-ORF8^∆ARKSAP^ (Fig. 1H). The extreme instability of ORF8 in the nucleus further excludes the possibility that ORF8 can act as a histone mimic.

We also reinvestigated whether ORF8 regulates H3 PTMs. We did not observe significant alternation in the levels of H3K9me3 and H3K27me3 upon ORF8 expression using immunofluorescence (Fig. 1I and 1J). ORF8 expression slightly decreased H3K9ac staining compared with mock-transfected cells (*P* = 0.0129), and ORF8^∆ARKSAP^ further decreased the H3K9ac level, suggesting that such a change was not due to histone mimicry (Fig. 1K). Similarly, no significant changes in H3 PTMs upon ORF8 expression were detected by immunoblotting (Fig. 1L). The changes on H3 PTMs induced by ORF8 reported by Kee *et al*. were quite small [fig. 2b–h and Supplementary fig. 1e in Kee *et al*. (2022)]. Taken together, these results support our suspicion that ORF8 alone is not sufficient to disrupt chromatin regulation. Additional evidence that does not support ORF8 as a histone mimic comes from their virus infection experiments. A mutant version of SARS-CoV-2 lacking the ARKS motif of ORF8, SARS-CoV-2^ΔARKSAP^, exhibited similar, and in some cases even higher, titers measured by plaque assays compared with WT SARS-CoV-2 [figs. 3b, 4f, and Supplementary table 4 in Kee *et al*. (2022)], and only slightly affected viral genome copy number [figs. 3a and 4e in Kee *et al*. (2022)]. The authors interpreted that ORF8 may promote the formation of viral particles in an ARKS-independent manner, such as in ER stress, which is actively involved in viral replication and modulates the innate host responses to the virus (Fung and Liu 2014; Xue and Feng 2021). Thus, we determined the effect of ORF8^∆ARKSAP^ on ER stress by monitoring the UPR pathways. ORF8^∆ARKSAP^ significantly elevated the phosphorylation of IRE1α and the level of its downstream substrate spliced XBP1 (XBP1s). ORF8^∆ARKSAP^ also elevated the phosphorylation of eIF2α, a substrate of the PERK branch. In addition, ORF8^∆ARKSAP^ induced the expression of BiP, a key ER chaperone involved in SARS-CoV-2 infection (Ha *et al*. 2020; Kohli *et al*. 2021; Shin *et al*. 2022). Importantly, the strength of the UPR was induced to a similar extent by ORF8^∆ARKSAP^ and WT ORF8 (Fig. 1M), which may at least partially explain why SARS-CoV-2^ΔARKSAP^ and WT virus did not show much difference in viral replication.

In conclusion, as a unique protein with extremely low sequence conservation among coronaviruses, ORF8 exerts multiple functions and is viewed as an important virulence factor of SARS-CoV-2 pathogenicity (Vinjamuri *et al*. 2022). However, the effects of ORF8 are less likely attributed to histone mimicry, because ORF8 is an ER lumen protein but not in the nucleus. In addition, the amount of escaped ORF8, if any, is not sufficient considering that histones are among the most abundant proteins in eukaryotic cells (Guo *et al*. 2018). Instead, the effect observed by Kee *et al*. is likely to due to other unknown mechanisms. The activation of UPR is known to involve many epigenetic changes, including chromatin modifications, gene expression, activation of transcription factors, and regulation of noncoding RNAs (Barroso and Chevet 2016). Thus, epigenetic regulation may be a downstream effect mediated by the ORF8-induced UPR, and this possibility requires further investigation. Alternatively, epigenetic disruption is a complex and synergistic effect orchestrated by ORF8 and other viral proteins. Further studies on the mechanisms of how ORF8 alters virus infection and spread would warrant novel intervention strategies against SARS-CoV-2.

## Supplementary Material

pwad042_suppl_Supplementary_Materials

## Footnotes

We thank Chih-chen Wang, Xi Wang, Zhuqiang Zhang, Zheng Zhou, Bing Zhu, Chunyan Fan, and Wei Ji (Institute of Biophysics, CAS) for helpful discussion, and Duo Duo, Xing Jia, and Qing Bian (Center for Biological Imaging, Institute of Biophysics, CAS) for technical support with FV3000 confocal imaging, LSM980 Airyscan imaging, and image analysis.

This work was supported by the National Key R&D Program of China (2021YFA1300800, 2022YFA1303000), the National Natural Science Foundation of China (Grant Nos. 92254305, 32271204, 32022033, and 32000872), the Strategic Priority Research Program of CAS (XDB37020303), the Project for Young Scientists in Basic Research of CAS (YSBR-075), and the Youth Innovation Promotion Association, CAS, to L.W.

J.H., and L.W. conceived the study. P.L. and L.W. designed the experiments. P.L. performed the experiments and data analyses. P.L., J.H., and L.W. wrote the manuscript.
