# Supplementary material for: SARS-CoV-2 ORF8 does not function in the nucleus as a histone mimic
Source: Protein Cell. 2023 Jul 14;15(2):79–82. doi: 10.1093/procel/pwad042 (PMC10833455; doi:10.1093/procel/pwad042)
Supplement: pwad042_suppl_Supplementary_Materials [file pwad042_suppl_supplementary_materials.pdf]

SARS-CoV-2 ORF8 does not function in the nucleus as a histone mimic

Materials and methods

Plasmids

pcDNA6B-FLAG plasmid encoding SARS-CoV-2 ORF8 was reported previously (Liu et al., 2022). cDNA encoding $ORF8^{\Delta ARKSAP}$ with deletion of the ARKSAP motif was cloned into pcDNA6B-FLAG vector. cDNAs encoding NLS-ORF8 and $NLS\text{-}ORF8^{\Delta ARKSAP}$ were generated by replacing the signal peptide sequence of ORF8 with the sequence encoding the NLS of SV40 large T-antigen (CCAAAGAAGAAGCGGAAGGTC).

Cell culture and transfection

HEK293T cells were cultured in Dulbecco's Modified Eagle's Medium (DMEM, Hyclone) supplemented with 10% fetal bovine serum (Gibco), 100 μg/ml streptomycin, and 100 units/ml penicillin (Invitrogen) at 37 ℃ in 5% CO_2. Transfections were performed using Lipofectamine 3000 (Invitrogen) according to the manufacturer's protocol.

Immunofluorescence

Treated cells cultured on glass bottom dishes (Cellvis) were washed with PBS three times, fixed with 4% paraformaldehyde (PFA) for 15 min, and permeabilized with 0.3% Triton X-100 for 5 min. After blocking with 5% BSA for 1 h at room temperature, cells were incubated with corresponding primary antibodies at 4 ℃ overnight and then with fluorescent-conjugated secondary antibodies for 1 h in the dark. The cells were rinsed with PBS and analyzed by a FV3000RS confocal microscope (Olympus) and LSM 980 with Airyscan 2 (Zeiss).

Immunoblotting

Cells were harvested and lysed in RIPA lysis buffer (50 mM Tris-HCl, pH 7.4, 150 mM NaCl, 0.25% deoxycholic acid, 1% NP-40, 1 mM EDTA) (Millipore) with phosphatase and protease inhibitor cocktails (Roche) on ice for 30 min. The supernatant was collected after centrifugation at 17,000 × *g* for 20 min. The protein concentration was quantified using a BCA protein assay kit (Beyotime). The samples were loaded and analyzed by SDS-PAGE and

transferred onto a polyvinylidene fluoride (PVDF) membrane. The membranes were blocked with 5% (w/v) nonfat milk or bovine serum albumin in TBST (50 mM Tris-HCl, pH 8.0, 150 mM NaCl, 0.05% Tween-20) buffer, incubated with antibodies, and visualized by a ChemiScope Mini imaging system (Clinx Science).

Subcellular fractionation

HEK293T cells expressing ORF8-FLAG or ORF8$^{\Delta ARKSAP}$-FLAG were lysed with cytoplasmic lysis buffer (10 mM HEPES, pH 7.9, 10 mM KCl, 1.5 mM $MgCl_2$, 1 mM DTT, 0.4% (v/v) NP-40, and protease inhibitor cocktail) on ice for 15 min; the supernatant (cytoplasmic fraction) was collected by centrifugation at 10,000 × *g* for 3 min. The intact nuclei were washed twice with cytoplasmic lysis buffer and then lysed with nuclear lysis buffer (20 mM HEPES, pH 7.9, 0.1 mM EGTA, 0.2 mM EDTA, 420 mM NaCl, 1 mM DTT, 25% (v/v) glycerol, 0.1% (v/v) NP-40, and protease inhibitor cocktail) with vortexing for 30 min at 4 ℃. The supernatant (nuclear fraction) was collected by centrifugation at 12,000 × *g* for 15 min.

PCR and sequencing

Total RNA was isolated from transfected cells using TRIzol (Invitrogen), and then 3 μg total RNA was reverse transcribed into cDNA using the GoScript Reverse Transcription System (Promega). cDNA of ORF8 and its mutants was amplified by PCR using the primers for pcDNA6B-FLAG: T7 promoter (TAATACGACTCACTATAGGG) and BGH reverse (TAGAAGGCACAGTCGAGG). The products were analyzed on a 2% agarose gel and visualized by SYBR Gold (Invitrogen) staining. For sequence determination, PCR products were purified using a DNA gel extraction kit (Omega) and subjected to Sanger sequencing using the above primers.

Quantification and statistical analysis

Graph plots and *p*-values were generated using GraphPad Prism 7 software (Graphpad). The density of immunoblot bands was quantified using ImageJ software (NIH). Data were presented as the mean ± SD. One-way ANOVA followed by Tukey's multiple comparison test was used to compare the means of more than two groups, and the *p*-values are indicated in the figures.

Data availability

All other data supporting the findings of this study are available from the corresponding author upon request.

Table 1. List of the sources and identifiers of antibodies and chemicals

REAGENT or RESOURCE	SOURCE	IDENTIFIER
**Antibodies**		
Mouse monoclonal anti-FLAG	Sigma-Aldrich	Cat#F1804;<br>RRID: AB_262044
Rabbit monoclonal anti-FLAG	Sigma-Aldrich	Cat#F7425;<br>RRID: AB_439687
Mouse monoclonal anti-PDI	Abcam	Cat#ab2792;<br>RRID: AB_303304
Rabbit polyclonal anti-Histone H3K9me3	Active Motif	Cat#39162
Rabbit polyclonal anti- Histone H3K27me3	Active Motif	Cat#39157
Mouse monoclonal anti-Histone H3K9ac	Active Motif	Cat#61252
Rabbit polyclonal anti-Histone H3	Abcam	Cat#ab1791;<br>RRID: AB_302613
Rabbit monoclonal anti-IRE1α	Cell Signaling Technology	Cat#3294S;<br>RRID: AB_823545
Rabbit monoclonal anti-pS724-IRE1α	Abcam	Cat#ab124945;<br>RRID: AB_11001365
Rabbit monoclonal anti-XBP1s	Cell Signaling Technology	Cat#12782S;<br>RRID: AB_2687943
Rabbit monoclonal anti-eIF2α	Cell Signaling Technology	Cat#5324S;<br>RRID: AB_10692650
Rabbit monoclonal anti-pS51-eIF2α	Cell Signaling Technology	Cat#9721S;<br>RRID: AB_330951
Rabbit polyclonal anti-GRP78/BiP	Sigma-Aldrich	Cat#G8918;<br>RRID: AB_477030
Mouse monoclonal anti-Calnexin	Millipore	Cat#MABF2067
Rabbit polyclonal anti-Lamin B1	Abcam	Cat#ab16048;<br>RRID: AB_443298
Anti-rabbit IgG-HRP	Sigma-Aldrich	Cat#A0545;<br>RRID: AB_257896
Anti-mouse IgG-HRP	Sigma-Aldrich	Cat#A4416;<br>RRID: AB_258167
Alexa Fluor 488 goat anti-rabbit IgG	Invitrogen	Cat#A27034;<br>RRID: AB_2536097
Alexa Fluor 568 goat anti-mouse IgG	Invitrogen	Cat#A11004;<br>RRID: AB_2534072
Alexa Fluor 488 mouse anti-mouse IgG	Invitrogen	Cat#A28175;<br>RRID: AB_2536161

Alexa Fluor 568 donkey anti-rabbit IgG	Invitrogen	Cat#A10042; RRID: AB_2534017
Mouse monoclonal anti-β-actin	Sigma-Aldrich	Cat#A3854; RRID: AB_262011
**Chemicals**		
Bafilomycin A1	Cell Signaling Technology	Cat#54645
MG132	Absin	Cat#abs817874
Hoechst 33258	Sigma-Aldrich	Cat#94403

References

Liu, P., Wang, X., Sun, Y., Zhao, H., Cheng, F., Wang, J., Yang, F., Hu, J., Zhang, H., Wang, C.C., *et al*. SARS-CoV-2 ORF8 reshapes the ER through forming mixed disulfides with ER oxidoreductases. Redox Biol 2022; 54: 102388.
